# Contribution of accumulative affective problems across the life course towards the association of childhood socioeconomic position with later-life cognitive ageing

**DOI:** 10.1007/s10433-026-00912-1

**Published:** 2026-03-10

**Authors:** Anouk Geraets, Sarah-Naomi James, Yiwen Liu, Marcus Richards, Anja Leist

**Affiliations:** 1 Department of Social Sciences, University of Luxembourg, Esch-Sur-Alzette, Luxembourg; 2 MRC Unit for Lifelong Health and Ageing, University College London, London, UK

**Keywords:** Socioeconomic position, Cognition, Affective problems, Cohort

## Abstract

Socioeconomic inequalities may act on affective and cognitive health from early life through later life. This study tested to what extent the association of childhood socioeconomic position (SEP) with later-life cognitive ageing is explained by life course accumulative affective problems. We used data from the MRC National Survey of Health and Development (*n* = 1,593; 52.6% women). Cognitive ageing was assessed through a battery of neuropsychological tests. Later-life cognitive function was assessed at age 69, and cognitive decline was measured across ages 53, 60–64, and 69. Childhood SEP was derived from overcrowding, essential household amenities, housing condition, and paternal occupation. Affective problems measured at ages 13–64 were categorized into case-level thresholds (never/once/twice or more). Multiple linear and logistic regression analyses and causal mediation models tested associations between childhood SEP, affective problems, and later-life cognitive ageing. Low childhood SEP was associated with lower scores on later-life (age 69) verbal memory (B = − 1.87[− 2.48; − 1.25]), letter search speed (B = − 9.98[− 17.57; − 2.40]), letter search accuracy (B = − 0.90[− 1.44; − 0.37]), cognitive state (B = − 2.14[− 2.82; − 1.46]), and accelerated decline (between ages 53–69) in letter search accuracy (B = 0.82[0.17; 1.46]), but slower decline in verbal memory (B = − 0.27[− 0.51; − 0.00]) compared to high childhood SEP. Low childhood SEP was associated with life course affective problems once (OR = 1.37[1.09; 1.73]) and twice or more (OR = 1.32[1.01; 1.71]). The association between childhood SEP and later-life letter search accuracy was partly explained (6.3%) by incidental affective problems. In conclusion, although improving affective health across the life course may enhance later-life cognitive ageing, it could only marginally explain the association between childhood SEP and later-life cognitive ageing.

**Supplementary Information:**

The online version contains supplementary material available at 10.1007/s10433-026-00912-1.

## Introduction

Social inequalities in cognitive ageing have been reported, in which more deprived individuals are at higher risk of cognitive impairment and dementia (Bodryzlova et al. 2022; Wang et al. 2023). Socioeconomic position (SEP) may provide cognitive stimulation (Beck et al. 2018), material opportunities (Baker 2014), and prestige (Hughes et al. 2024), which may act on cognitive function from early life through to older ages (Beck et al. 2018; Geraets et al. 2024; Liu et al. 2023; McElroy et al. 2021). Alternatively, SEP may lead to cognitive ageing through affective problems. In contrast to clinical diagnoses of depressive disorders, affective problems refer to the broader range of mood-related symptoms that capture both subclinical mood symptoms and clinical disorders and are often measured through questionnaires. Both low childhood and adult SEP are related to an increased risk of affective problems (Angelini et al. 2018; Patel et al. 2018). In addition, associations between SEP and affective problems are suggested to be stronger for persistent affective problems compared to incidental affective problems (Lorant et al. 2003), which might be due to the often persistent nature of low SEP (Cohen et al. 2010). Depression is among the top modifiable risk factors for dementia included in the 2024 report on dementia of the Lancet commission (Livingston et al. 2024), with meta-analyses showing a two times higher dementia risk (Diniz et al. 2013). Though the association between depression and dementia is complex (Bennett & Thomas 2014; Brzezińska et al. 2020), the risk of cognitive impairment and dementia is suggested to be stronger for recurrent affective problems compared to incidental affective problems (Dotson et al. 2010; James et al. 2018; Kessing & Andersen 2004). Exposure to accumulative affective problems across the life course may affect the brain through multiple biological pathways, including the autonomic, cardiometabolic, and immune system (McEwen & Gianaros 2010).

The contribution of accumulative affective problems across the life course to the association between childhood SEP and later-life cognitive ageing remains unclear. A recent study has shown that later-life debt and poverty were associated with an increased risk of cognitive impairment and dementia, and that these associations were up to 30% mediated by concurrent affective problems (Zhou et al. 2021). However, whether this mediation becomes stronger for accumulative affective problems across the life course, and whether these associations already begin in childhood, remains unknown. Data on life course SEP, affective problems, and cognitive function are scarce. The Medical Research Council (MRC) National Survey of Health and Development (NSHD), the British 1946 birth cohort, has collected data on SEP, affective problems, and cognitive function over multiple time points, which provides valuable insights into factors for cognitive ageing across the life course (Richards 2022). Previous research of the NSHD cohort has shown that father’s occupation in childhood was associated with later-life cognitive function independent of adult SEP (Hurst et al. 2013). Furthermore, previous NSHD research has shown that accumulative affective problems across the life course predict poorer cognitive function in midlife (John et al. 2019a, b) and later life (James et al. 2018). Lastly, John et al. (2021a, b) found an association of later-life affective symptoms with verbal memory and processing speed, but verbal memory and processing speed could not predict subsequent later-life affective symptoms in the NSHD cohort. All these findings from the NSHD cohort support a contributing role of accumulative affective problems on the association between childhood SEP and later-life cognitive ageing.

**Fig. 1:**
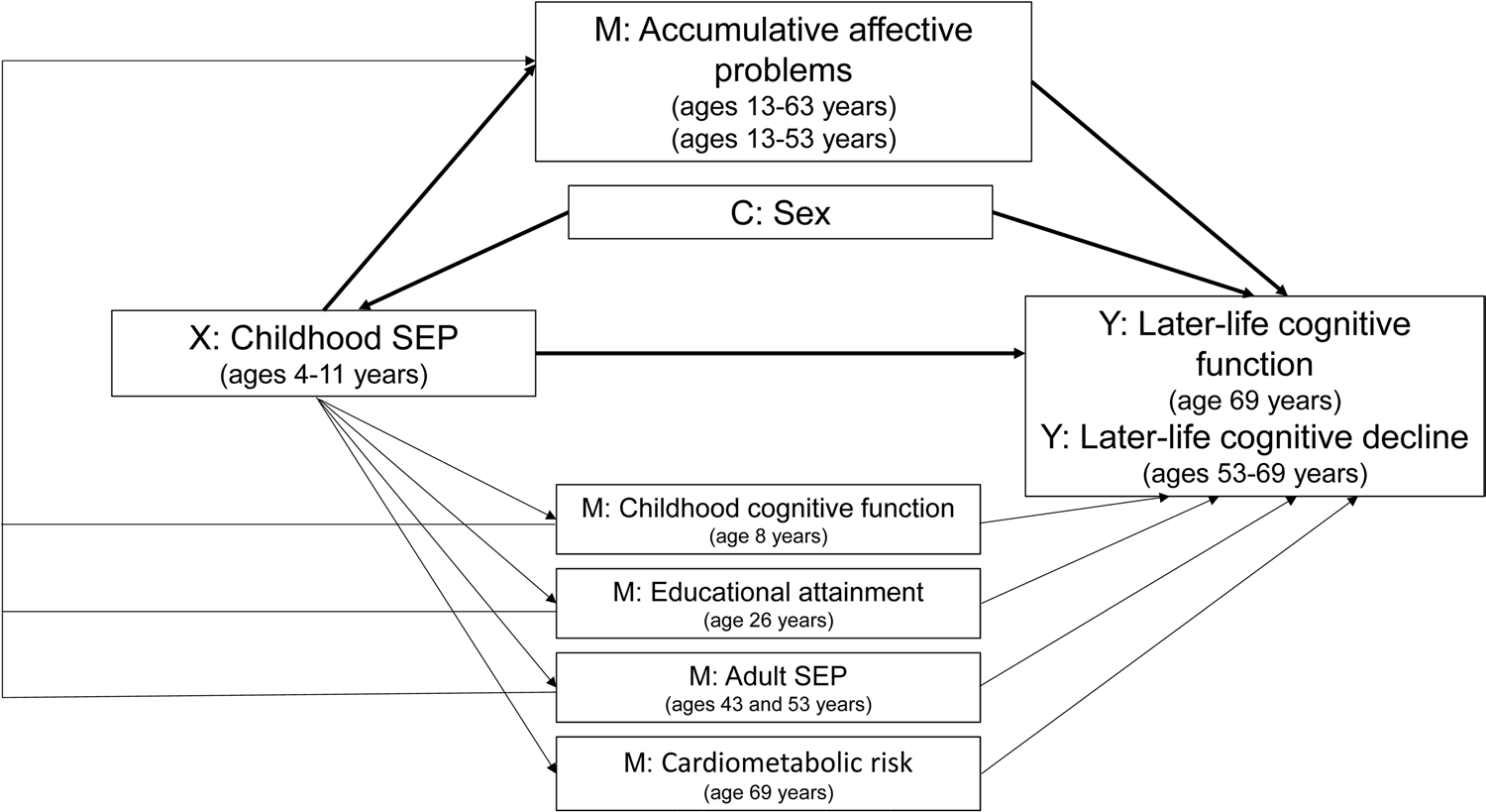
Directed acyclic graph reflecting assumed relationships between childhood socioeconomic position, accumulative affective problems, and later-life cognition. X indicates exposure; C, baseline confounder; M, mediator; Y, outcome; SEP, socioeconomic position

The aim of this study was to test the hypotheses that (1) lower childhood SEP and accumulative affective problems contribute to lower later-life cognitive function and accelerated cognitive decline, and (2) accumulative affective problems partly explain the association between lower childhood SEP and later-life cognitive ageing (Fig. 1).

## Methods

### Study population and design

**Fig. 2:**
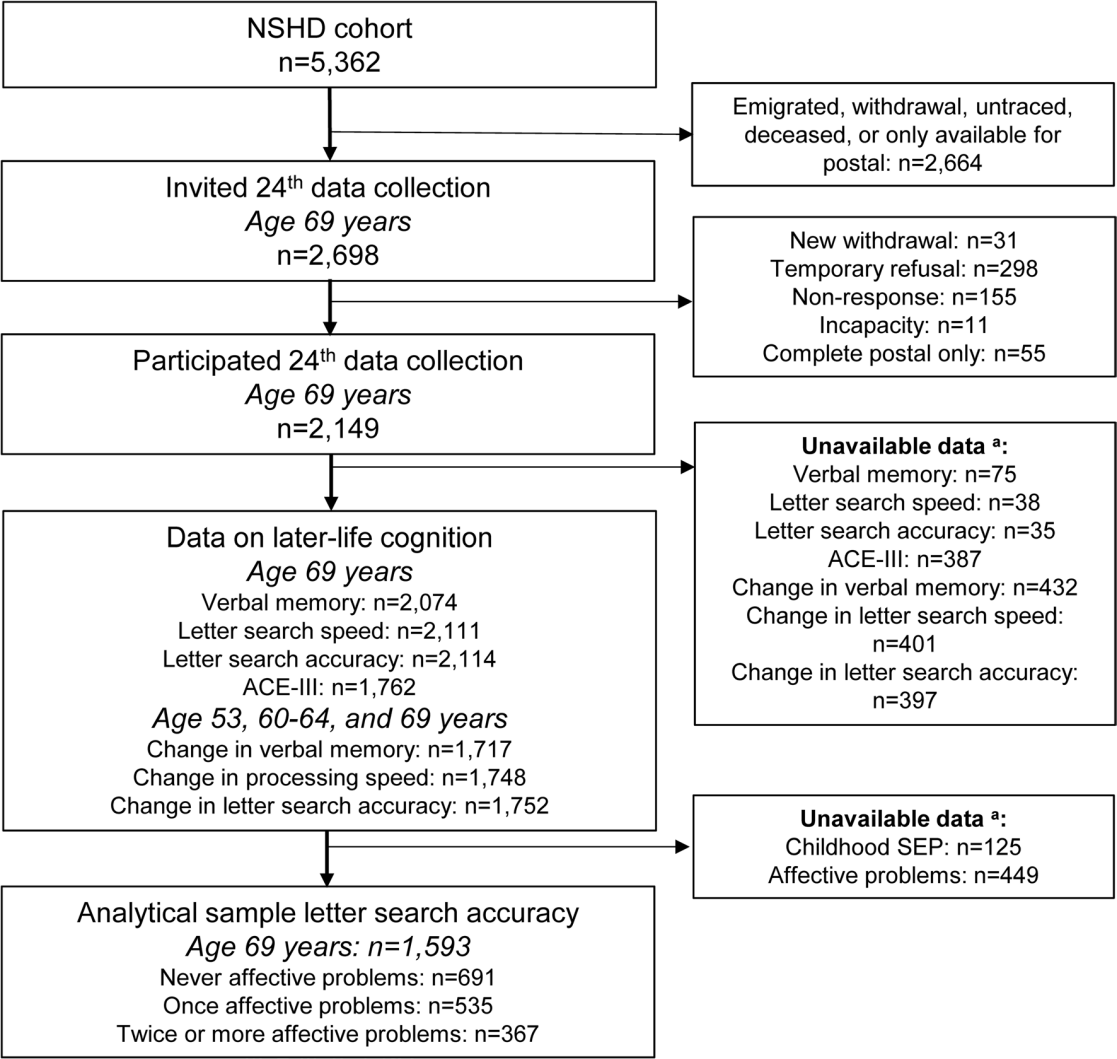
Flowchart of the study population. ^a^Numbers are not mutually exclusive

Data were from the Medical Research Council (MRC) National Survey of Health and Development (NSHD), the British 1946 birth cohort. This cohort originally consisted of a representative sample of 5,362 males and females who were born in England, Scotland, and Wales in one week in March 1946 (Wadsworth et al. 2006). The flowchart of the study population is shown in Fig. 2. At age 69 years, 2,149 participants participated in the 24th data collection during a home visit (Kuh et al. 2016), of whom 2,074 participants had data on short-term verbal memory, 2,111 on letter search speed, 2,114 on letter search accuracy, and 1,762 on cognitive state. In addition, 1,717, 1748, and 1,752 participants also had data on, respectively, verbal memory, letter search speed, and letter search accuracy at ages 53 and 60–64 years. Data on childhood SEP and life course affective problems were unavailable in, respectively, 125 and 449 participants. As missing cognition data at age 69 years were not missing at random (eTable 1), to prevent losing statistical power, and to respect the time and efforts participants have spent in their assessments, we decided to exploit all available data for the individual cognitive outcomes, resulting in analytical samples ranging from 1,593 for letter search accuracy to 1,335 for cognitive state.

The authors assert that all procedures contributing to this work comply with the ethical standards of the relevant national and institutional committees on human experimentation and with the Helsinki Declaration of 1975, as revised in 2013. All procedures involving human subjects/patients were approved by the NRES Queen Square Research Ethics Committee (14/LO/1073) and Scotland A Research Ethics Committee (14/SS/1009). All participants gave written informed consent.

### Measurements

#### Childhood socioeconomic position

Childhood SEP was derived from overcrowding (≥ 2 people per room) at age 4, lack of essential household amenities (no sole use of kitchen or bathroom, or no running hot water) at age 4 or age 11 years, below average housing condition rated by the interviewer at age 4 years (excluding those who lived in a caravan), and paternal occupational status (unskilled or unemployed, reported at age 11, 4, or 15 years). The presence of any of these adversities reflects low childhood SEP, in line with previous work (Liu et al. 2023). To increase statistical efficiency and to reduce variation between each SEP indicator, a composite score for childhood SEP was created indicating high or low childhood SEP (respectively absence or presence of any indicator of economic adversity).

#### Life course affective problems

Due to the nature of data collection across the life course, different assessments of affective problems were applied from adolescence to late life. As described previously, ‘case-level symptoms’ were identified in those with a level of symptom severity consistent with a possible clinical diagnosis of affective disorder at each age (James et al. 2018). At ages 13 and 15, teacher ratings of behaviour and temperament were obtained using a forerunner of the Adolescent Rutter scale (Rutter 1967). Factor scores at ages 13 and 15 were summed and standardized to create scales representing a dimension of emotional problems (case-level symptoms: 91st–100th percentile) (Colman et al. 2007). Affective problems in adulthood were measured with the short community version of the Present State Examination at age 36 (case-level symptoms: score ≥ 5) (Rodgers & Mann 1986; Wing et al. 1974), the Psychiatric Symptom Frequency scale at age 43 (case-level symptoms: score ≥ 23) (Lindelow et al. 1997), and the 28-item General Health Questionnaire (case-level symptoms: score ≥ 5) (Goldberg & Hillier 1979) at age 53 and 60–64. In addition, participants were classified as having affective problems at ages 36, 43, 53, and 60–64 if they used anxiolytic (British National Formulary Sect. 4.1.2) or antidepressant (British National Formulary Sect. 4.3) medication (yes/no) (Association 1999). The number of times participants had affective problems across testing waves was summed and recoded as follows: (a) never (no incomplete date on affective problems allowed); (b) once only (incomplete date on affective problems allowed); (c) twice or more (incomplete date on affective problems allowed). For analyses on cognitive change (between ages 53 and 69 years), a similar variable was created that excluded case-level affective problems at age 60–64 years.

#### Cognition

Short-term verbal memory and processing speed were assessed at ages 53, 60–64, and 69. Short-term verbal memory was assessed using a 15-item word learning task devised by the NSHD. The total number of words correctly recalled over three identical trials was summed to provide an overall score for short-term verbal memory (Richards et al. 2014). Processing speed was assessed by a visual search task, where participants were required to cross out the letters P and W, randomly embedded within a page of other letters, as quickly and accurately as possible within 1 min. Letter search speed was represented by the position reached at the end of this interval (maximum 600), and letter search accuracy was represented by the number of target letters correctly crossed out within this interval (maximum 84). In addition, cognitive state at age 69 was assessed with a customized version of the Addenbrooke's Cognitive Examination III (ACE-III) (Hsieh et al. 2013) by iPad using ACEMobile (http://www.acemobile.org) or if not possible by paper as the primary outcome measure of later-life cognition. The ACE-III (score range 0–100) is divided into five domains: attention and orientation (scored 0–18); verbal fluency (0–14); memory (0–26); language (0–26); and visuospatial function (0–16).

#### Covariates

Consistent with previous analyses, all analyses were adjusted for sex as potential confounder (Liu et al. 2023). Childhood cognitive function, educational attainment, adult SEP (defined by occupational position and household income), and cardiometabolic health factors are considered mediators, as childhood SEP may be related to later-life cognition via lower childhood cognitive function (Liu et al. 2023), educational attainment and adult SEP (Beck et al. 2018; Geraets et al. 2024; John et al. 2022; Liu et al. 2023; McElroy et al. 2021), and a worse later-life cardiometabolic risk profile (including coronary heart disease, cerebrovascular disease, kidney disease, higher waist circumference, higher systolic blood pressure, higher haemoglobin A1c level, higher triglyceride-to-HDL ratio, smoking, higher alcohol consumption, and lower physical activity) (John et al. 2021a, b) (Fig. 1). Therefore, these factors (described in more detail in the Supplemental methods) were included as covariates in sensitivity analyses.

### Statistical analyses

Analyses were conducted in Stata 18.0 (StataCorp, College Station, TX, USA). Participants with available cognition data at age 69 had a higher childhood SEP, less accumulative affective problems, a higher educational attainment, and a healthier cardiometabolic risk profile compared to those without these data (eTable 1). Consequently, there was a selection bias in which the association between childhood SEP and affective problems disappeared in this healthier study sample with available cognition data at age 69 (eTable 2). To reduce bias arising from this non-random missingness of available cognition data at age 69, we constructed unstabilized inverse probability weights based on the estimated likelihood of being included in the analytic sample. A logistic regression model predicting availability of cognition data at age 69 was performed using childhood SEP, accumulative affective problems, and sex as predictors. For all participants with observed cognition data at age 69, we calculated a weight equal to the inverse of the predicted probability of being observed $({w}_{i}=1/{\widehat{p}}_{i})$. Participants with a low estimated probability received a higher weight, and those with a high estimated probability received a lower weight. These weights were then applied in all subsequent analyses.

Differences in general characteristics between the childhood SEP groups were tested using survey-weighted linear regression accounting for probability weights (continuous variables) or Rao–Scott adjusted tests (ordinal and dichotomous variables). Mixed effect regression models were applied to assess cognitive decline in short-term verbal memory and processing speed from age 53 to 69. A quadratic term for cognitive change over time was used and models were fitted with random intercepts and slopes with unstructured covariance matrices. The slopes of these unadjusted estimates were used to measure later-life cognitive decline. Baseline cognition scores (at age 53) were included in the models investigating change in cognitive function, in line with recommendations for statistical analyses that aim to derive causal conclusions from observational data (Tennant et al. 2022).

To test hypothesis 1, multivariable linear regression analyses were conducted to investigate the associations of childhood SEP and affective problems with later-life cognitive function (age 69) and cognitive decline (age 53–69). Associations between childhood SEP and affective problems were tested using binary logistic regression analyses in which affective problems were entered as dummy variables and affective problems once and twice or more were compared to never affective problems in different models.

To test whether affective problems explained the association between childhood SEP and later-life cognitive ageing (hypothesis 2), we used the ‘mediate’ command in Stata, which performs causal mediation analysis within a counterfactual framework (StataCorp 2023). Probit models were used to estimate the total effect of childhood SEP on later-life cognitive ageing and to decompose its total effects into direct and indirect effects via affective problems. A two-sided *p*-value of 0.05 was applied.

### Additional analyses

Several sensitivity analyses were performed. First, associations of the individual childhood SEP indicators with, respectively, cognitive outcomes and affective problems were tested. To investigate whether associations concern fluid cognitive functions (general cognitive abilities that do not depend on prior knowledge), we additionally adjusted for the National Adult Reading Test score (Nelson & Willison 1991; Richards & Sacker 2003) as a proxy measure of cognitive intelligence. Use of anxiolytic or antidepressant medication could have led to remission. Therefore, participants using these medications were removed from analyses in sensitivity analyses. As childhood cognition, educational attainment, adult SEP, and cardiometabolic and behavioural factors may be on the causal pathway from SEP to affective problems and cognitive ageing (John et al. 2021a, b; Liu et al. 2023; Opdebeeck et al. 2016; Richards et al. 2014), we additionally adjusted for these potential mediators in additional analyses (Fig. 1). Lastly, we tested whether there were life course sensitive periods for the associations of childhood SEP with case-level affective problems in the total study population with or without cognition data at age 69 years, as previous research has found stronger associations for affective problems after age 53 with verbal memory and ACE-III score, and affective problems up to age 53 with letter search speed and accuracy score (James et al. 2018).

## Results

Table 1 shows the general characteristics of the weighted study population with data on letter search accuracy stratified by SEP as this concerned the largest analytical sample (*n* = 1,593). Participants with a low childhood SEP were more likely to have had affective problems across the life course, lower cognition scores in later life, and a worse cardiometabolic risk profile compared to those with a high childhood SEP. Case-level affective problems and antidepressant medication use per measurement are presented in eTable 3.

**Table 1:** General characteristics of the weighted study population stratified by childhood SEP

	High childhood SEP * n* = 651	Low childhood SEP * n* = 942
Characteristic		
Demographics		
Sex, n (% female)	474 (52.7)	873 (54.3)
Educational attainment, n (% low)	**399 (45.5)**	**1,112 (71.3)**
Composite score adult SEP, n (% low)	**784 (87.5)**	**1,192 (75.1)**
Childhood SEP (ages 4–11 years)		
Overcrowding, n (%)	**0 (0)**	**419 (26.6)**
Lack of essential household amenities, n (%)	**0 (0)**	**1,447 (90.1)**
Below average housing condition, n (%)	**0 (0)**	**161 (10.4)**
Paternal occupation, n (%)	**0 (0)**	**171 (10.9)**
Childhood cognitive function (age 8 years)		
Standardized summary cognitive score, mean (SD)	**0.34 (0.03)**	− **0.01 (0.03)**
Accumulative affective problems		
Life course affective problems (ages 13–63 years), never/once/twice or more, n (%)	**408/334/133 (46.6/38.2/15.2)**	**620/682/253 (39.9/43.8/16.3)**
Life course affective problems (ages 13–53 years), never/once/twice or more, n (%)	**330/354/215 (36.7/39.4/24.0)**	**475/714/420 (29.5/44.4/26.1)**
Later-life cognitive decline (ages 53–69 years)		
Change in verbal memory, mean (SD)	**2.9 (0.2)**	**1.5 (0.2)**
Change in letter search speed, mean (SD)	− 4.0 (3.7)	− 11.2 (3.0)
Change in letter search accuracy, mean (SD)	1.1 (0.6)	− 0.0 (0.5)
Later-life cognitive function (age 69 years)		
Verbal memory score, mean (SD)	**23.1 (0.2)**	**21.3 (0.2)**
Letter search speed score, mean (SD)	**266.7 (3.0)**	**256.7 (2.4)**
Letter search accuracy score, mean (SD)	**24.0 (0.2)**	**23.1 (0.2)**
ACE-III total score, mean (SD)	**92.7 (0.2)**	**90.4 (0.2)**
Later-life fluid cognitive function (age 53 years)		
Score National Adult Reading Test, mean (SD)	**12.3 (0.3)**	**16.2 (0.3)**
Later-life cardiometabolic risk factors (age 69 years)		
Coronary heart disease, n (%)	80 (9.0)	164 (10.3)
Cerebrovascular disease, n (%)	34 (3.9)	86 (5.5)
Kidney disease, n (%)	13 (1.5)	43 (2.7)
Waist circumference (cm), mean (SD)	**95.4 (0.5)**	**97.2 (0.5)**
Systolic blood pressure (mm/Hg), mean (SD)	133.3 (0.7)	134.0 (0.6)
Haemoglobin A1c (%), mean (SD)	5.81 (0.03)	5.83 (0.02)
Triglyceride-to-HDL ratio, mean (SD)	1.25 (0.04)	1.29 (0.04)
Later-life behavioural factors (age 69 years)		
Smoking, never/former/current, n (%)	254/563/65 (28.8/63.8/7.4)	478/959/159 (30.0/60.1/10.0)
Frequency alcohol use (times per week), 0–1/2–3/ > 3, n (%)	**331/233/301 (38.3/26.9/34.7)**	**770/356/394 (50.6/23.4/25.9)**
Any physical activity last week, n (%)	**669 (83.2)**	**1,113 (78.1)**

Numbers are provided for the weighted study population with data on letter search accuracy as this concerns the largest analytical sample (*n* = 1,593). Data are presented as estimated means ± linearized standard error or weighted counts (%)

Statistically significant associations using a two-sided *p*-value < 0.05 are presented in bold

SEP indicates socioeconomic position, *SD* standard deviation, *ACE-III* Addenbrooke's Cognitive Examination III, *HDL* high-density lipoprotein

### Association of childhood SEP and accumulative affective problems with cognitive ageing

Low childhood SEP compared to high childhood SEP was associated with lower scores on later-life (age 69) verbal memory (unstandardized regression coefficient [B] = − 1.87[− 2.48; − 1.25]), letter search speed (B = − 9.98[− 17.57; − 2.40]), letter search accuracy (B = − 0.90[− 1.44; − 0.37]), cognitive state (B = − 2.14[− 2.82; − 1.46]), and accelerated decline in letter search accuracy (B = 0.82[0.17; 1.46]). A slower decline in verbal memory (B = − 0.27[− 0.51; − 0.03]) between age 53 and 69 was observed for those with a low childhood SEP, while no association was found between low childhood SEP and change in letter search speed score (B = 2.98[− 0.52; 6.47]; Table 2).

**Table 2:** Associations of childhood socioeconomic position and accumulative affective problems with later-life cognition

Model	Verbal memory score (*n* = 1,568)	Letter search speed score (*n* = 1,591)	Letter search accuracy score (*n* = 1,593)	ACE-III score (*n* = 1,335)	Change in verbal memory score (*n* = 1,402)	Change in letter search speed score (*n* = 1,423)	Change in letter search accuracy score (*n* = 1,426)
	B (95% CI)	B (95% CI)		B (95% CI)	B (95% CI)	B (95% CI)	
Childhood SEP							
High	Reference	Reference	Reference	Reference	Reference	Reference	Reference
Low	− **1.87 (**− **2.48; **− **1.25)**	− **9.98 (**− **17.57; **− **2.40)**	− **0.90 (**− **1.44; **− **0.37)**	− **2.14 (**− **2.82; **− **1.46)**	− **0.27 (**− **0.51; **− **0.03)**	2.98 (− 0.52; 6.47)	**0.82 (0.17; 1.46)**
Accumulative affective problems							
Never	Reference	Reference	Reference	Reference	Reference	Reference	Reference
Once	− **0.81 (**− **1.48; **− **0.14)**	− 8.36 (− 16.81; 0.08)	− **0.98 (**− **1.58; **− **0.39)**	− **1.04 (**− **1.77; **− **0.30)**	− 0.18 (− 0.43; 0.07)	3.31 (− 0.47; 7.09)	0.50 (− 0.19; 1.18)
Twice or more	− **1.26 (**− **2.08; **− **0.43)**	− **14.19 (**− **23.68; **− **4.69)**	− **1.23 (**− **1.88; **− **0.58)**	− **1.90 (**− **2.82; **− **0.98)**	0.31 (− 0.06; 0.68)	2.07 (− 3.34; 7.47)	0.42 (− 0.54; 1.39)

Analyses are adjusted for sex and childhood cognitive function. Analyses including change are additionally adjusted for baseline score. Statistically significant associations using a two-sided *p*-value < 0.05 are presented in bold

ACE-III indicates Addenbrooke's Cognitive Examination III, *B* unstandardized regression coefficient, *CI* confidence interval, *SEP* socioeconomic position

Affective problems, both once and twice or more across the life course, were associated with lower scores on verbal memory (B = − 0.81[− 1.48; − 0.14] and B = − 1.26[− 2.08; − 0.43], respectively), letter search accuracy (B = − 0.98[− 1.58; − 0.39] and B = − 1.23[− 1.88; − 0.58], respectively), and cognitive state at age 69 years (B = − 1.04[− 1.77; − 0.30] and B = − 1.90[− 2.82; − 0.98], respectively; Table 2). In addition, twice or more affective problems were associated with letter search speed at age 69 years (B = − 14.19[− 23.68; − 4.69]). No associations between affective problems and change in cognitive function were found (Table 2).

### Associations of childhood SEP with accumulative affective problems

Participants with a low childhood SEP were more likely to have affective problems once (odds ratio [OR] = 1.37[1.09; 1.73]) or twice or more (OR = 1.32[1.01; 1.71]) compared to participants with a high childhood SEP (eTable 2).

### Mediation analyses

As we only found associations of both childhood SEP and affective problems with later-life cognitive function at age 69 years, we only decomposed the total effect of childhood SEP on cognitive test scores at age 69 years into direct and indirect effects via, respectively, accumulative affective problems once and twice or more (Table 3). There was an indirect effect for affective problems once on letter search speed and letter search accuracy (B = − 0.07[− 0.14; − 0.00]), in which 6.3% of the total effect of low childhood SEP on letter search accuracy score could be attributed to its relation with affective problems once. No other significant indirect effects were found (Table 3).

**Table 3:** Decomposed associations of childhood socioeconomic position with later-life cognitive function via accumulative affective problems

Model	Verbal memory score	Letter search speed score	Letter search accuracy score	ACE-III score
	B (95% CI)	B (95% CI)	B (95% CI)	B (95% CI)
**Once affective problems**				
**Low childhood SEP **^**a**^				
Direct	− **1.93 (**− **2.62; **− **1.25)**	− **12.11 (**− **20.76; **− **3.45)**	− **1.04 (**− **1.65; **− **0.43)**	− **2.10 (**− **2.80; **− **1.39)**
Indirect via once affective problems ^b^	− 0.05 (− 0.12; 0.01)	− 0.56 (− 1.34; 0.22)	− **0.07 (**− **0.14; **− **0.00)**	− 0.06 (− 0.13; 0.01)
Total	− **1.99 (**− **2.68; **− **1.30)**	− **12.67 (**− **21.36; **− **3.98)**	− **1.11 (**− **1.72; **− **0.50)**	− **2.16 (**− **2.86; **− **1.45)**
**Twice or more affective problems**				
**Low childhood SEP **^**a**^				
Direct	− **1.60 (**− **2.35; **− **0.85)**	− 9.01 (− 18.10; 0.08)	− 0.57 (− 1.21; 0.06)	− **2.21 (**− **3.03; **− **1.39)**
Indirect via ≥ 2 affective problems ^b^	− 0.08 (− 0.18; 0.01)	− 1.06 (− 2.17; 0.05)	− 0.09 (− 0.18; 0.00)	− 0.12 (− 0.26; 0.02)
Total	− **1.69 (**− **2.45; **− **0.92)**	− **10.07 (**− **19.27; **− **0.88)**	− **0.66 (**− **1.30; **− **0.02)**	− **2.33 (**− **3.16; **− **1.50)**

*n* = 1,208 (verbal memory score and once affective problems), *n* = 1,223 (letter search speed score and once affective problems), *n* = 1,226 (letter search accuracy score and once affective problems), *n* = 1,007 (ACE-III score and once affective problems), *n* = 1,038 (verbal memory score and ≥ 2 affective problems), *n* = 1,057 (letter search speed score and ≥ 2 affective problems), *n* = 1,058 (letter search accuracy score and ≥ 2 affective problems), and *n* = 887 (ACE-III score and ≥ 2 affective problems)

Regression results are decomposed in direct, indirect, and total effects and presented as mean difference with 95% CI and adjusted for sex and childhood cognitive function. Statistically significant associations using a two-sided *p*-value < 0.05 are presented in bold

ACE-III indicates Addenbrooke's Cognitive Examination III, *B* unstandardized regression coefficient, *CI* confidence interval, *SEP* socioeconomic position

^a^Compared to high childhood SEP. ^b^Compared to no accumulative affective problems

### Additional analyses

Although some of the associations between the individual childhood SEP indicators with later-life cognitive function and affective problems were statistically non-significant, they were all in the same direction (eTables 4–5). The associations of childhood SEP with later-life cognitive ageing attenuated, and some of them became statistically non-significant after additional adjustment for childhood cognitive function, education, or adult SEP (eTable 6). Additional adjustment for cardiometabolic or behavioural factors did not materially change the associations between childhood SEP and later-life cognitive ageing (eTable 6). All associations between childhood SEP and later-life cognitive ageing attenuated and became statistically non-significant after additional adjustment for fluid cognitive function, while excluding participants that used anxiolytic or antidepressant medication strengthened the associations between childhood SEP and cognitive ageing (eTable 6). The association between childhood SEP and affective problems once did not materially change after additional adjustments, while associations between childhood SEP and affective problems twice or more became statistically non-significant after additional adjustments (eTable 7). However, estimates were only reduced by more than 10% after additional adjustment for childhood cognition, education, or adulthood SEP. Exclusion of participants that used anxiolytics or antidepressants strengthened the association between childhood SEP and affective problems twice or more (eTable 7). Lastly, stronger associations between childhood SEP and case-level affective problems during adolescence were reported compared to case-level affective problems during adulthood (eTable 8).

## Discussion

This study aimed to investigate the associations of childhood SEP, accumulative affective problems from adolescence to later life, and later-life cognitive ageing. We found that individuals with a lower childhood SEP had worse later-life short-term verbal memory, processing speed, and cognitive state and accelerated decline in later-life processing speed but slower decline in verbal memory compared to individuals with a high childhood SEP. Although we found that a lower childhood SEP was associated with affective problems across the life course, and that affective problems were associated with worse later-life cognitive function, incidental affective problems could only partially explain (6.3%) the effect of low childhood SEP on later-life processing speed.

Findings of this study are in line with previous findings that showed associations between lower childhood SEP and worse later-life cognitive function (Aartsen et al. 2019; Beck et al. 2018; Geraets et al. 2024; Liu et al. 2023; McElroy et al. 2021; Richards et al. 2019). We extend these findings by prospectively showing an accelerated decline in later-life processing speed among those with a lower childhood SEP, but a slower decline in later-life verbal memory among those with early life socioeconomic disadvantages. An accelerated decline in later-life cognitive function for those with a lower childhood SEP has been observed in the Health and Retirement Study (Oi & Haas 2019). However, this association operated entirely through adult SEP (Oi & Haas 2019). This corresponds to the recently published study by Krueger et al. (2025) that observed associations between lifetime SEP and change in later-life cognition but not between childhood SEP and later-life cognitive change independent of adult SEP. A slower verbal memory decline among those with lower childhood SEP has also been observed in the large Survey of Health, Aging, and Retirement in Europe (Aartsen et al. 2019). This might be due to higher cognitive reserve, the capacity of the brain to compensate for brain damage through efficient or flexible use of cognitive processes shaped by lifetime intellectual enrichment (Stern 2002). Those with a higher childhood SEP may have been more likely to have gained more cognitive reserve through higher educational and occupational attainment later in life, resulting in higher baseline cognition scores. These higher achievements accomplished through social exposures may be better reflected in higher baseline memory scores in later life, and consequently a steeper decline in memory function, compared to processing speed that may be considered as a more general measure of cognitive function more closely linked to biological processes. Important to note is that all these cohort studies measured childhood SEP retrospectively by parental occupational and/or educational attainment. This may not only have caused recall bias because of the time between childhood and the data collection (Althubaiti 2016), but results may also have been confounded by fluid intelligence that has genetically passed from parents to child (Kent 2017). Using life course data, we found that the associations between childhood SEP and later-life cognitive ageing attenuated after additional adjustment for childhood cognition, educational attainment, or verbal fluid intelligence, while additional adjustment for adult SEP measured by income and occupational attainment had a smaller influence on the estimates.

We observed associations between affective problems across the life course and later-life cognitive function but not later-life cognitive decline. These results reflect the divergence of previous research from the NSHD cohort by observing different associations of life course affective problems with later-life cognitive state versus later-life cognitive change. Earlier findings of associations between recurrent affective problems across the life course and lower later-life cognitive function (James et al. 2018) could not be extended to later-life changes in cognitive function. This is in line with previous work from John et al. (John et al. 2019a, b) that could not identify associations of affective problems in adolescence or adulthood (ages 36 and 43) with later-life cognitive decline (John et al. 2019a, b).

The contribution of affective problems across the life course on the negative effect of low childhood SEP on later-life cognitive function was marginal, as only incidental affective problems partly explained (6.3%) the effect of low childhood SEP on later-life processing speed. This is in contrast to the suggested large contribution (30%) of affective problems on socioeconomic inequalities in cognitive impairment and dementia in later life (Zhou et al. 2021). It might be that socioeconomic disadvantage only affects current affective and cognitive health. In this case, low childhood SEP associates with both affective and cognitive health in early life, as shown in previous studies (Hackman & Farah 2009; Larson et al. 2015; Reiss 2013), while its effect on later-life cognition is more strongly affected by SEP in later life. Indeed, it has been shown that the association between childhood SEP and later-life cognitive function is largely dependent on childhood cognitive function, childhood emotional problems, education, and later-life SEP (Beck et al. 2018; Geraets et al. 2024; Krueger et al. 2025; Liu et al. 2023; Luo & Waite 2005; McElroy et al. 2021). Furthermore, previous work from the NSHD cohort has shown that affective problems in adolescence but not in adulthood were related to later-life cognitive function (John et al. 2019a, b). These findings underscore the importance of life course models in the research on cognitive ageing.

This study has several strengths. We used data of the oldest British birth cohort that studied individual life courses over 70 years. This large homogenous sample allowed us to investigate multiple stages across the life course. All measures were assessed prospectively, which made it possible to longitudinally investigate the accumulation of affective problems over the life course and to measure changes in later-life cognition. Other studies mainly assess childhood factors retrospectively, which is a major limitation because of information biases (Althubaiti 2016). In addition, the use of measures over multiple time points provided us with the opportunity to look at life course sensitive periods. Lastly, the extensiveness of the data allowed us to include a broad range of sensitivity analyses to test the robustness of findings.

Certain limitations should be acknowledged as well. Inherent to cohort studies, there might have been selection and attrition bias, in which the most healthy individuals participate and remain in the study (Nunan et al. 2018). In our study, we found that the association between childhood SEP and affective problems attenuated and became statistically non-significant in the study sample with later-life cognition data. Although we weighted our analytical sample for missingness of later-life cognition data, these biases may have led to an underestimation of the associations between childhood SEP, affective problems, and later-life cognitive ageing. Second, although childhood SEP was captured by a composite score based on material conditions rather than relying exclusively on parental occupational status, the impact of these material conditions might have been mitigated by the fact that material deprivation was a common and shared experience rather than a minority stigma after World War II. In 1946, at the start of the study, food and clothing rationing was still present. More than half of the participants had no sole use of a kitchen or bathroom or no running hot water during childhood, which, however, changed quickly with the reconstruction processes in the 1950s. Third, childhood cognitive functioning was treated as a mediator between childhood SEP and later-life cognitive functioning, as cognitive abilities can be assumed to have little influence on parental SEP characteristics. However, both childhood cognitive functioning and parental SEP characteristics may be partly determined by parental cognitive abilities, which we could not control for in this study. Lastly, there was a lack of ethnic diversity. Previous studies have shown the importance of ethnicity and its intercorrelation with SEP on cognitive ageing (Glymour & Manly 2008; Krueger et al. 2025; Zahodne et al. 2021). More inclusive research is needed on whether minority groups may be disproportionately affected by the accumulation of affective problems across the life course to better understand inequalities in later-life cognitive ageing.

## Conclusions

In conclusion, early life socioeconomic disadvantages can have a negative impact on affective health across the life course and later-life cognitive ageing. Although affective problems across the life course may act on later-life cognitive ageing, they seem to act independently of childhood SEP, as they could only marginally explain the relation between childhood SEP and later-life cognition. Improving mental health across the life course may reduce inequalities in later-life cognitive ageing. However, the negative effects of low childhood SEP on later-life cognitive ageing may be better explained by education and adult SEP.

## Supplementary Information

Below is the link to the electronic supplementary material.

Supplementary file1 (DOCX 66 KB)

## Data Availability

The data that support the findings of this study are available to bona fide researchers upon request to the NSHD Data Sharing Committee. For more information, see: ().
