# Supplementary material for: Contribution of accumulative affective problems across the life course towards the association of childhood socioeconomic position with later-life cognitive ageing
Source: Eur J Ageing. 2026 Mar 10;23(1):16. doi: 10.1007/s10433-026-00912-1 (PMC13086983; doi:10.1007/s10433-026-00912-1)
Supplement: Supplementary file 1 — Supplementary file1 (DOCX 66 KB) [file 10433_2026_912_MOESM1_ESM.docx]

**Supplemental Material**

**Contribution of accumulative affective problems across the life course towards the association of childhood socioeconomic position with later-life cognitive ageing**

Anouk Geraets, Ph.D.^1^*, Sarah-Naomi James, Ph.D.^2^, Yiwen Liu, Ph.D.^2^, Marcus Richards, Ph.D.^2^, Anja Leist, Ph.D.^1^

^1^ Department of Social Sciences, University of Luxembourg, Esch-Sur-Alzette, Luxembourg

^2^ MRC Unit for Lifelong Health and Ageing, University College London, London, United Kingdom

**Supplemental methods**

**Measurements covariates**

Childhood cognitive function at age 8 was measured as the sum of four tests of verbal and non-verbal ability devised by the National Foundation for Educational Research standardized to the whole population (Pigeon 1964).

Educational attainment by age 26 years was classified as no qualification, vocational, below ordinary secondary qualifications, ordinary level qualifications (‘O’ levels or their training equivalents), advanced level qualifications (‘A’ levels or their equivalents), above advanced level qualification, degree, Masters, or doctorate and dichotomized into those with advanced qualification or higher vs. those below this level.

Adult SEP was assessed with occupational position and household income. Occupational position was derived at age 53, given that this is when most people are expected to be in work, or earlier than this if information was missing. Occupational position was dichotomized into manual vs. non-manual. Net household income represented income after deduction for income tax, national insurance, state benefits and other income sources, and included contributions from other household members. The bottom 20% of the annual net household income assessed at age 43 and 53 years was classified as low (score=0) and compared to the upper 80% in the analytical sample (score=1). The average of these income measure was classified as low (score=0 or 0.5) or moderate/high (score=1). A composite adult SEP was created indicating high or low adult SEP (absence or presence of any indicator of economic adversity).

Waist circumference (cm), systolic blood pressure (mm/Hg), hemoglobin A1c (%), triglycerides, and HDL cholesterol at age 69 were assessed as previously described (Pierce et al. 2012). Smoking status (current smoker/ex-smoker/never smoked), frequency of alcohol use (≤1 per week/2-3 times per week/≥4 times per week), involvement in physical activity over the past week (yes/no), coronary heart disease (heart failure, angina, myocardial infarction) cerebrovascular disease (stroke, transient ischemic attacks), and kidney disease at age 69 were self-reported.

**Supplemental tables**

**eTable 1. General characteristics of participants with and without cognition data at age 69 years**

| Characteristic | Cognition data (n=2,131) | No cognition data (n=3,231) | *p*-value |
| --- | --- | --- | --- |
| Demographics |  |  |  |
| Sex, n (% female) | 1,086 (51.0) | 1,460 (45.2) | <0.001 |
| Educational attainment, n (% low) | 1,197 (59.3) | 1,783 (73.9) | <0.001 |
| Composite score adult SEP, n (% low) | 1,704 (81.4) | 1,297 (76.4) | <0.001 |
| Childhood SEP (ages 4-11 years) |  |  |  |
| Composite score childhood SEP, n (% low) | 1,180 (58.8) | 1,939 (67.0) | <0.001 |
| Overcrowding, n (%) | 309 (15.3) | 579 (22.0) | <0.001 |
| Lack of essential household amenities, n (%) | 1,063 (51.2) | 1,733 (59.5) | <0.001 |
| Below average housing condition, n (%) | 128 (6.4) | 238 (9.1) | 0.001 |
| Paternal occupation, n (%) | 125 (6.2) | 254 (9.5) | <0.001 |
| Accumulative affective problems |  |  |  |
| Life course affective problems (ages 13-63 years), never/once/twice or more, n (%) | 705/577/396  (42.0/34.4/23.6) | 116/564/283  (12.1/58.6/29.4) | <0.001 |
| Life course affective problems (ages 13-53 years), never/once/twice or more, n (%) | 928/566/241  (53.5/32.6/13.9) | 349/544/249  (30.6/47.6/21.8) | <0.001 |
| Later-life fluid cognitive function (age 53 years) |  |  |  |
| Score National Adult Reading Test, mean (SD) | 18.6 (10.1) | 14.4 (9.0) | <0.001 |
| Late-life cardiometabolic risk factors (age 69 years) |  |  |  |
| Coronary heart disease, n (%) | 196 (9.2) | 47 (62.7) | <0.001 |
| Cerebrovascular disease, n (%) | 85 (4.0) | 23 (44.2) | <0.001 |
| Kidney disease, n (%) | 49 (2.3) | 1 (1.6) | 0.718 |
| Waist circumference (cm), mean (SD) | 96.5 (13.9) | 101.8 (14.9) | 0.155 |
| Systolic blood pressure (mm/Hg), mean (SD) | 133.6 (16.8) | 127.8 (13.6) | 0.161 |
| Haemoglobin A1c (%), mean (SD) | 5.81 (0.69) | 5.53 (0.28) | 0.193 |
| Triglyceride-to-HDL ratio, mean (SD) | 1.27 (1.00) | 0.90 (0.55) | 0.222 |
| Late-life behavioural factors (age 69 years) |  |  |  |
| Smoking, never/former/current, n (%) | 645/1,275/184  (30.7/60.6/8.8) | 148/284/63  (29.9/57.4/12.7) | 0.168 |
| Frequency alcohol use (times per week), 0-1/2-3/>3, n (%) | 895/515/620  (44.1/25.4/30.5) | 8/3/2  (61.5/23.1/15.4) | 0.169 |
| Any physical activity last week, n (%) | 1,511 (80.7) | 6 (75.0) | 0.683 |

Data are presented as means ± standard deviation or number (%) and differences between those with and without cognition data are tested by chi-squared tests (binary variables), Mann-Whitney U tests (ordinal variables), or t-tests (continuous variables). SEP indicates socioeconomic position; SD, standard deviation; HDL, high-density lipoprotein.

**eTable 2. Associations of childhood socioeconomic position with accumulative affective problems in in different subsamples**

| Model | Once affective problems ^a^ |  | Twice or more affective problems ^a^ |  |
| --- | --- | --- | --- | --- |
|  | **OR (95% CI)** | **n=** | **OR (95% CI)** | **n=** |
| Total study population |  | 1,884 |  | 1,456 |
| Childhood SEP |  |  |  |  |
| High | Reference |  | Reference |  |
| Low | **1.30 (1.08;1.58)** |  | **1.45 (1.16;1.81)** |  |
| Study population with cognition data at age 69 years unweighted |  | 1,234 |  | 1,066 |
| Childhood SEP |  |  |  |  |
| High | Reference |  | Reference |  |
| Low | 1.12 (0.89;1.42) |  | 1.11 (0.86;1.45) |  |
| Study population with cognition data at age 69 years weighted |  | 1,234 |  | 1,066 |
| Childhood SEP |  |  |  |  |
| High | Reference |  | Reference |  |
| Low | **1.37 (1.09;1.73)** |  | **1.32 (1.01;1.71)** |  |

Total study population n=1,884 and n=1,456; with data on letter search accuracy n=1,234 and n=1,066 for respectively once and twice or more affective problems. OR indicates odds ratio; CI, confidence interval; SEP, socioeconomic position. Analyses are adjusted for sex. Statistically significant associations using a two-sided p-value < 0.05 are presented in bold. ^a^ Reference category is no accumulative affective problems.

**eTable 3. Affective problems per measurement in the study population with data on letter search accuracy (n=1,593)**

| Affective problems | n (%) | Missing, n (%) |
| --- | --- | --- |
| Adult affective problems (ages 36-63 years), never/once/twice or more, n (%) | 691/535/367  (43.4/33.6/23.0) | 0 (0.0) |
| Adult affective problems (ages 36-53 years), never/once/twice or more, n (%) | 787/466/301  (49.4/29.3/18.9) | 39 (2.5) |
| Affective problems age 13 years, n (%) | 135 (8.5) | 80 (5.0) |
| Affective problems age 15 years, n (%) | 134 (8.4) | 93 (5.8) |
| Affective problems age 36 years, n (%) | 137 (8.6) | 69 (4.3) |
| Affective problems according questionnaire age 36 years, n (%) | 94 (5.9) | 69 (4.3) |
| Antidepressant or anxiolytic medication use age 36 years, n (%) | 63 (4.0) | 67 (4.2) |
| Affective problems age 43 years, n (%) | 252 (15.8) | 33 (2.1) |
| Affective problems according questionnaire age 43 years, n (%) | 217 (13.6) | 33 (2.1) |
| Antidepressant or anxiolytic medication use age 43 years, n (%) | 68 (4.3) | 32 (2.0) |
| Affective problems age 53 years, n (%) | 402 (25.2) | 48 (3.0) |
| Affective problems according questionnaire age 53 years, n (%) | 352 (22.1) | 49 (3.1) |
| Antidepressant or anxiolytic medication use age 53 years, n (%) | 88 (5.5) | 34 (2.1) |
| Affective problems age 63 years, n (%) | 401 (25.2) | 83 (5.2) |
| Affective problems according questionnaire age 63 years, n (%) | 292 (18.3) | 98 (6.2) |
| Antidepressant or anxiolytic medication use age 63 years, n (%) | 154 (9.7) | 47 (3.0) |

Data are presented as number (%).

**eTable 4. Associations of individual indicators of childhood socioeconomic position with-later life cognition**

| Model | Verbal memory score | Letter search speed score | Letter search accuracy score | ACE-III score | Change in verbal memory score | Change in letter search speed score | Change in letter search accuracy score |
| --- | --- | --- | --- | --- | --- | --- | --- |
|  | **B (95% CI)** | **B (95% CI)** | **B (95% CI)** | **B (95% CI)** | **B (95% CI)** | **B (95% CI)** | **B (95% CI)** |
| Overcrowding (n=1,576) ^a^ |  |  |  |  |  |  |  |
| No | Reference | Reference | Reference | Reference | Reference | Reference | Reference |
| Yes | **-2.12(-3.03;-1.21)** | **-12.66 (-22.89;-2.42)** | **-1.48 (-2.19;-0.77)** | **-2.88 (-3.91;-1.86)** | -0.04 (-0.35;0.27) | 0.87 (-3.95;5.69) | **1.20 (0.34;2.06)** |
| Lack of essential household amenities (n=1,591) ^a^ |  |  |  |  |  |  |  |
| No | Reference | Reference | Reference | Reference | Reference | Reference | Reference |
| Yes | **-1.37 (-1.98;-0.76)** | **-7.70 (-15.19;-0.22)** | **-0.69 (-1.21;-0.16)** | **-1.52 (-2.21;-0.83)** | **-0.24 (-0.47;-0.00)** | 3.05 (-0.47;6.57) | 0.62 (-0.01;1.25) |
| Below average housing condition (n=1,568) ^a^ |  |  |  |  |  |  |  |
| No | Reference | Reference | Reference | Reference | Reference | Reference | Reference |
| Yes | **-2.28 (-3.51;-1.04)** | -11.40 (-26.06;3.26) | **-1.33 (-2.36;-0.30)** | -1.24 (-2.64;0.16) | -0.44 (-0.91;0.03) | 0.04 (-6.71;6.78) | 0.75 (-0.61;2.12) |
| Low paternal occupation (n=1,571) ^a^ |  |  |  |  |  |  |  |
| No | Reference | Reference | Reference | Reference | Reference | Reference | Reference |
| Yes | **-2.22 (-3.40;-1.04)** | -5.49 (-18.41;7.44) | **-0.96 (-1.85;-0.07)** | **-2.85 (-4.42;-1.28)** | -0.32 (-0.82;0.18) | 0.09 (-6.74;6.91) | **1.15 (0.13;2.16)** |

B indicates unstandardized regression coefficient; CI, confidence interval; SEP, socioeconomic position; ACE-III, Addenbrooke's Cognitive Examination III. Analyses are adjusted for sex and analyses including change are additionally adjusted for baseline score. Statistically significant associations using a two-sided p-value < 0.05 are presented in bold.^a^ Numbers are provided for letter search accuracy score.

**eTable 5. Associations of individual indicators of childhood socioeconomic position with accumulative affective problems**

| Model | Once affective problems ^a^ |  | Twice or more affective problems ^a^ |  |
| --- | --- | --- | --- | --- |
|  | **OR (95% CI)** | **n=** | **OR (95% CI)** | **n=** |
| Overcrowding |  | 1,212 |  | 1,051 |
| No | Reference |  | Reference |  |
| Yes | 1.26 (0.92;1.74) |  | 1.13 (0.78;1.64) |  |
| Lack of essential household amenities |  | 1,225 |  | 1.057 |
| No | Reference |  | Reference |  |
| Yes | 1.24 (0.99;1.56) |  | 1.17 (0.90;1.52) |  |
| Below average housing condition |  | 1,209 |  | 1.046 |
| No | Reference |  | Reference |  |
| Yes | 1.02 (0.62;1.67) |  | 1.13 (0.67;1.93) |  |
| Low paternal occupation |  | 1,209 |  | 1,043 |
| No | Reference |  | Reference |  |
| Yes | 1.50 (0.93;2.41) |  | 1.29 (0.74;2.25) |  |

OR indicates odds ratio; CI, confidence interval; SEP, socioeconomic position. Analyses are adjusted for sex. Statistically significant associations using a two-sided p-value < 0.05 are presented in bold. ^a^ Reference category is no accumulative affective problems.

**eTable 6**. **Additional analyses of childhood socioeconomic position with later-life cognition**

| Model | Verbal memory score | Letter search speed score | Letter search accuracy score | ACE-III score | Change in verbal memory score | Change in letter search speed score | Change in letter search accuracy score |
| --- | --- | --- | --- | --- | --- | --- | --- |
|  | **B (95% CI)** | **B (95% CI)** | **B (95% CI)** | **B (95% CI)** | **B (95% CI)** | **B (95% CI)** | **B (95% CI)** |
| Model 1: childhood SEP (n=1,593) ^a^ |  |  |  |  |  |  |  |
| High | Reference | Reference | Reference | Reference | Reference | Reference | Reference |
| Low | **-1.87 (-2.48;-1.25)** | **-9.98 (-17.57;-2.40)** | **-0.90 (-1.44;-0.37)** | **-2.14 (-2.82;-1.46)** | **-0.28 (-0.52;-0.04)** | 3.05 (-0.49;6.60) | **0.80 (0.16;1.44)** |
| Model 1 + childhood cognition (n=1,499) ^a^ |  |  |  |  |  |  |  |
| High | Reference | Reference | Reference | Reference | Reference | Reference | Reference |
| Low | **-0.81 (-1.39;-0.23)** | -6.42 (-14.54;1.70) | -0.47 (-1.04;0.09) | **-0.80 (-1.42;-0.18)** | -0.17 (-0.41;0.07) | 2.60 (-1.22;6.43) | 0.48 (-0.19;1.15) |
| Model 1 + education (n=1,550) ^a^ |  |  |  |  |  |  |  |
| High | Reference | Reference | Reference | Reference | Reference | Reference | Reference |
| Low | **-0.83 (-1.43;-0.23)** | -5.21 (-13.07;2.65) | -0.49 (-1.05;0.07) | **-1.08 (-1.75;-0.41)** | -0.07 (-0.31;0.17) | 2.67 (-1.04;6.37) | 0.50 (-0.16;1.17) |
| Model 1 + adult SEP (n=1,580) ^a^ |  |  |  |  |  |  |  |
| High | Reference | Reference | Reference | Reference | Reference | Reference | Reference |
| Low | **-1.36 (-1.96;-0.75)** | **-8.73 (-16.51;-0.95)** | **-0.69 (-1.23;-0.15)** | **-1.62 (-2.28;-0.97)** | -0.24 (-0.48;0.00) | 3.28 (-0.39;6.95) | 0.65 (-0.00;1.30) |
| Model 1 + cardiometabolic factors ^b^ (n=1,381) ^a^ |  |  |  |  |  |  |  |
| High | Reference | Reference | Reference | Reference | Reference | Reference | Reference |
| Low | **-1.69 (-2.35;-1.04)** | **-8.77 (-16.66;-0.87)** | **-0.87 (-1.43;-0.32)** | **-1.93 (-2.67;-1.19)** | -0.25 (-0.51;0.00) | 3.17 (-0.48;6.81) | **0.91 (0.24;1.57)** |
| Model 1 + behavioral factors ^c^ (n=1,340) ^a^ |  |  |  |  |  |  |  |
| High | Reference | Reference | Reference | Reference | Reference | Reference | Reference |
| Low | **-1.76 (-2.41;-1.11)** | **-11.44 (-19.78;-3.11)** | **-0.94 (-1.52;-0.36)** | **-1.88 (-2.58;-1.18)** | **-0.30 (-0.56;-0.05)** | 3.56 (-0.37;7.48) | **0.82 (0.12;1.52)** |
| Model 1 + NART score (n=1,511) ^a^ |  |  |  |  |  |  |  |
| High | Reference | Reference | Reference | Reference | Reference | Reference | Reference |
| Low | -0.48 (-1.05;0.09) | -6.95 (-14.78;0.87) | -0.40 (-0.96;0.15) | -0.48 (-1.10;0.14) | -0.09 (-0.33;0.14) | 2.58 (-1.09;6.25) | 0.40 (-0.25;1.05) |
| Model 1 excl. anxiolytics/antidepressants users (n=1,215) ^a^ |  |  |  |  |  |  |  |
| High | Reference | Reference | Reference | Reference | Reference | Reference | Reference |
| Low | **-1.50 (-2.19;-0.82)** | **-14.87 (-23.31;-6.44)** | **-1.12 (-1.72;-0.52)** | **-1.83 (-2.57;-1.09)** | **-0.26 (-0.52;-0.00)** | **4.43 (0.63;8.24)** | **0.91 (0.20;1.62)** |

ACE-III indicates Addenbrooke's Cognitive Examination III; B, unstandardized regression coefficient; CI, confidence interval; SEP, socioeconomic position; NART, National Adult Reading Test. Statistically significant associations using a two-sided p-value < 0.05 are presented in bold. All analyses are adjusted for sex and analyses including change are additionally adjusted for baseline score. ^a^ Numbers are provided for study population for letter search accuracy. ^b^ Includes coronary heart disease, cerebrovascular disease, kidney disease, waist circumference, systolic blood pressure, hemoglobin A1c, and triglyceride-to-HDL ratio. ^c^ Includes smoking status, alcohol use, and any physical activity.

**eTable 7. Additional analyses of the associations of childhood socioeconomic position with accumulative affective problems**

| Model | Once affective problems ^a^  (n=540) |  | Twice or more affective problems ^a^  (n=372) |  |
| --- | --- | --- | --- | --- |
|  | **OR (95% CI)** | **n=** | **OR (95% CI)** | **n=** |
| Model 1: childhood SEP |  | 1,234 |  | 1,066 |
| High | Reference |  | Reference |  |
| Low | **1.37 (1.09;1.73)** |  | **1.32 (1.01;1.71)** |  |
| Model 1 + childhood cognition |  | 1,164 |  | 1,013 |
| High | Reference |  | Reference |  |
| Low | **1.38 (1.08;1.75)** |  | 1.23 (0.94;1.62) |  |
| Model 1 + education |  | 1,198 |  | 1,042 |
| High | Reference |  | Reference |  |
| Low | **1.34 (1.05;1.70)** |  | 1.16 (0.89;1.53) |  |
| Model 1 + adult SEP |  | 1,225 |  | 1,062 |
| High | Reference |  | Reference |  |
| Low | **1.36 (1.07;1.72)** |  | 1.25 (0.96;1.64) |  |
| Model 1 + NART score |  | 1,171 |  | 1,027 |
| High | Reference |  | Reference |  |
| Low | **1.42 (1.11;1.81)** |  | 1.29 (0.98;1.69) |  |
| Model 1 excl. anxiolytics/antidepressants users |  | 1,066 |  | 852 |
| High | Reference |  | Reference |  |
| Low | **1.33 (1.03;1.72)** |  | 1.41 (0.99;2.03) |  |

OR indicates odds ratio; CI, confidence interval; SEP, socioeconomic position; NART, National Adult Reading Test. Analyses are adjusted for sex. Statistically significant associations using a two-sided p-value < 0.05 are presented in bold. ^a^ Reference category is no accumulative affective problems (n=694).

**eTable 8. Life course sensitive periods for the association between childhood socioeconomic position and case-level affective problems in study population with and without later-life cognition data**

| Model | Affective problems age 13 years  (n=4,021) | Affective problems age 15 years  (n=3,963) | Affective problems age 36 years  (n=3,137) | Affective problems age 43 years  (n=3,068) | Affective problems age 53 years  (n=2,754) | Affective problems age 63 years  (n=2,111) |
| --- | --- | --- | --- | --- | --- | --- |
|  | **OR (95% CI)** | **OR (95% CI)** | **OR (95% CI)** | **OR (95% CI)** | **OR (95% CI)** | **OR (95% CI)** |
| Childhood SEP |  |  |  |  |  |  |
| High | Reference | Reference | Reference | Reference | Reference | Reference |
| Low | **1.45 (1.15;1.82)** | **1.29 (1.03;1.62)** | 1.08 (0.83;1.40) | 1.19 (0.97;1.48) | 1.14 (0.95;1.38) | 1.08 (0.88;1.32) |

OR indicates odds ratio; CI, confidence interval; SEP, socioeconomic position. Analyses are adjusted for sex. Statistically significant associations using a two-sided p-value < 0.05 are presented in bold.

**References**

Pierce, M. B., Silverwood, R. J., Nitsch, D., Adams, J. E., Stephen, A. M., Nip, W., Macfarlane, P., Wong, A., Richards, M., & Hardy, R. (2012). Clinical disorders in a post war British cohort reaching retirement: evidence from the first national birth cohort study. PLoS One. <https://doi.org/10.1371/journal.pone.0044857>

Pigeon, D. (1964). Tests used in the 1954 and 1957 surveys. The home and the school 129-132.
